# Trimmomatic: a decade of feature-rich, high-performance NGS read preprocessing

**DOI:** 10.1093/bioinformatics/btag331

**Published:** 2026-05-22

**Authors:** Sebastian Beier, Anthony M Bolger, Marie E Bolger, Rainer Schwacke, Björn Usadel

**Affiliations:** Institute of Bio- and Geosciences (IBG-4: Bioinformatics), CEPLAS, BIOSC, Forschungszentrum Jülich, Wilhelm Johnen Straße, Jülich, 52425, Germany; Institute of Bio- and Geosciences (IBG-4: Bioinformatics), CEPLAS, BIOSC, Forschungszentrum Jülich, Wilhelm Johnen Straße, Jülich, 52425, Germany; Institute of Bio- and Geosciences (IBG-4: Bioinformatics), CEPLAS, BIOSC, Forschungszentrum Jülich, Wilhelm Johnen Straße, Jülich, 52425, Germany; Institute of Bio- and Geosciences (IBG-4: Bioinformatics), CEPLAS, BIOSC, Forschungszentrum Jülich, Wilhelm Johnen Straße, Jülich, 52425, Germany; Institute of Bio- and Geosciences (IBG-4: Bioinformatics), CEPLAS, BIOSC, Forschungszentrum Jülich, Wilhelm Johnen Straße, Jülich, 52425, Germany; Faculty of Mathematics and Natural Sciences, Institute for Biological Data Science, CEPLAS, Heinrich-Heine University Düsseldorf, Universitätsstr. 1, Düsseldorf, 40225, Germany

## Abstract

**Motivation:**

Trimmomatic is a widely adopted tool for preprocessing high-throughput sequencing data, particularly from Illumina platforms. Since its original publication in 2014, the volume and complexity of sequencing data have increased dramatically, necessitating continuous tool evolution.

**Results:**

We present the substantial updates to Trimmomatic over the past decade. Key enhancements include a robust multithreading model for high-performance parallel processing, parallel GZIP/BZIP2 compression, and a suite of new trimming and filtering steps to provide users with more flexible quality control. Usability has been significantly improved through automatic PHRED encoding detection and simplified file handling. The codebase has also been modernized including Maven support, and continuous integration to ensure long-term sustainability and community contributions. These updates solidify Trimmomatic’s role as an efficient, flexible, and essential tool in modern bioinformatics pipelines.

**Availability:**

Trimmomatic remains open-source under the GPL V3 license, with the latest version available at https://github.com/usadellab/Trimmomatic and also on our website https://www.plabipd.de/trimmomatic_main.html (DOI: https://doi.org/10.5281/zenodo.18678155).

## 1 Introduction

The preprocessing of next-generation sequencing (NGS) data to remove adapter sequences and low-quality regions is a necessary first step for reliable downstream analysis. In 2014, we introduced Trimmomatic, a flexible and efficient read trimmer that correctly handled paired-end data, a significant advancement at the time (Bolger *et al.* 2014). In the intervening years, Trimmomatic has been widely adopted by the bioinformatics community, becoming a staple tool in countless analysis pipelines and cited over 50 000 times (at the point of writing).

However, the NGS landscape has changed dramatically. The sheer scale of data has grown exponentially, with platforms like the Illumina NovaSeq series generating terabytes of data in a single run (Sboner *et al.* 2011, Goodwin *et al.* 2016). This explosion in volume means that processing, even for a “simple” step like trimming, can become a significant computational bottleneck. The original single-threaded architecture of Trimmomatic, while efficient for its time, is insufficient for these modern datasets.

Simultaneously, sequencing applications have diversified far beyond genomic resequencing. Analyses such as RNA-seq for gene expression (Wang *et al.* 2009), UMI-based protocols for low-input sequencing (Islam *et al.* 2014), and highly sensitive variant calling (Koboldt *et al.* 2013) all have unique and stringent requirements for data quality. These applications demand more granular and flexible control over trimming than the original toolset provided. For example, some protocols require simple, fixed-length truncation, while others benefit from more adaptive quality filtering methods.

Finally, the practice of bioinformatics itself has matured. Reproducibility, ease of installation, and integration into automated workflow management systems like Nextflow (Di Tommaso *et al.* 2017) or Snakemake (Köster and Rahmann 2012) are now primary concerns. Software from a decade ago, lacking modern build systems and continuous integration, creates a barrier to reproducible science and community-driven development (Grüning *et al.* 2018).

To address these challenges, Trimmomatic has undergone substantial evolution. Here, we describe the major enhancements implemented since its original publication, focusing on the critical additions of high-performance multithreading, expanded trimming capabilities, and essential software modernization to ensure Trimmomatic remains a fast, flexible, and sustainable tool for the next decade of sequencing.

## 2 New features and enhancements

To address the primary computational bottleneck of modern datasets, the core processing engine was adapted for concurrent operation. A high-performance multithreading architecture was implemented. This design allows batches of read pairs to be processed independently by a pool of worker threads, scaling efficiently with available hardware and dramatically reducing execution time. To ensure that file I/O does not become a new performance ceiling, this is complemented by parallel GZIP/BZIP2 compression. This utilizes separate threads for compression, preventing the main processing threads from stalling while writing compressed output to disk.

The trimming toolkit was also significantly expanded to provide more specialized operations required by modern protocols. For instance, the HEADCROP and TAILCROP steps were introduced to remove a set number of bases from the 5′ or 3′ end, respectively. This function is essential for handling reads containing Unique Molecular Identifiers (UMIs) or custom barcodes, as it allows for their precise removal to prevent interference with downstream alignment or variant calling. These steps also serve as a simple, fast method for fixed-length truncation.

Additional filtering methods were developed to provide more granular quality control. The AVGQUAL trimmer was added to discard entire reads if their average quality score falls below a user-defined threshold. This serves a different quality control philosophy than the existing SLIDINGWINDOW trimmer, which attempts to rescue reads by trimming low-quality regions. AVGQUAL, by contrast, is designed to quickly filter out reads that are globally poor, which is useful for data from sequencing cycles that experienced systemic failure. A new cleanup step, BASECOUNT, was also introduced to filter reads that fall below a specified length. Its purpose is to enforce a *post-trimming* length threshold, as aggressive trimming by other steps can leave fragments that are too short to be confidently or uniquely aligned (Li 2013), saving time in downstream analysis.

These new steps join the established MAXINFO adaptive quality trimmer, which remains a key feature for sensitive applications. This algorithm formalizes the trade-off between read length, which increases mappability, and the accumulating risk of errors from low-quality bases. It intelligently trims the read to the point that maximizes its informational content, providing a more robust alternative to simple windowing for applications like variant calling (Van der Auwera and O’Connor 2020) or *de novo* assembly (Prjibelski *et al.* 2020).

Usability and reliability in automated environments were also central to the new developments. To prevent a common and difficult-to-diagnose user error, automatic detection of the PHRED quality score encoding (Phred33 or Phred64) was implemented (Cock *et al.* 2010). An incorrect offset renders all quality-based trimming operations invalid, and this feature now removes that ambiguity. Furthermore, a built-in read pairing validator was added to check the integrity and order of paired-end files before processing begins. This ensures read pair synchronization is maintained, preventing data corruption in downstream pair-aware analyses. For pipeline integration, command-line syntax was simplified with template-based input and output file names, and reporting was enhanced with a summary statistics file for high-level quality control and an optional detailed trim log for fine-tuning trimming parameters.

Finally, the project’s software architecture and distribution methods were modernized to ensure long-term sustainability and accessibility. The codebase was updated to Java 8, and the build system was migrated from Ant to Maven to simplify dependency management and ensure consistent builds. Modern continuous integration (CI) routines and a suite of unit tests were established, providing a framework for automated validation that lowers the barrier for community contributions and ensures new features do not introduce regressions. To address the critical need for scientific reproducibility, Trimmomatic is now officially distributed through Bioconda (Grüning *et al.* 2018) and as both Docker and Singularity container images (Kurtzer *et al.* 2017), guaranteeing a consistent and easy-to-install environment for all users.

### 2.1 Performance at scale

To quantify the performance gains from the new architecture, a benchmark was performed. The experiment was designed to test two distinct features: the scalability of the multithreading engine and the impact of the new parallel compression.

**Figure 1:**
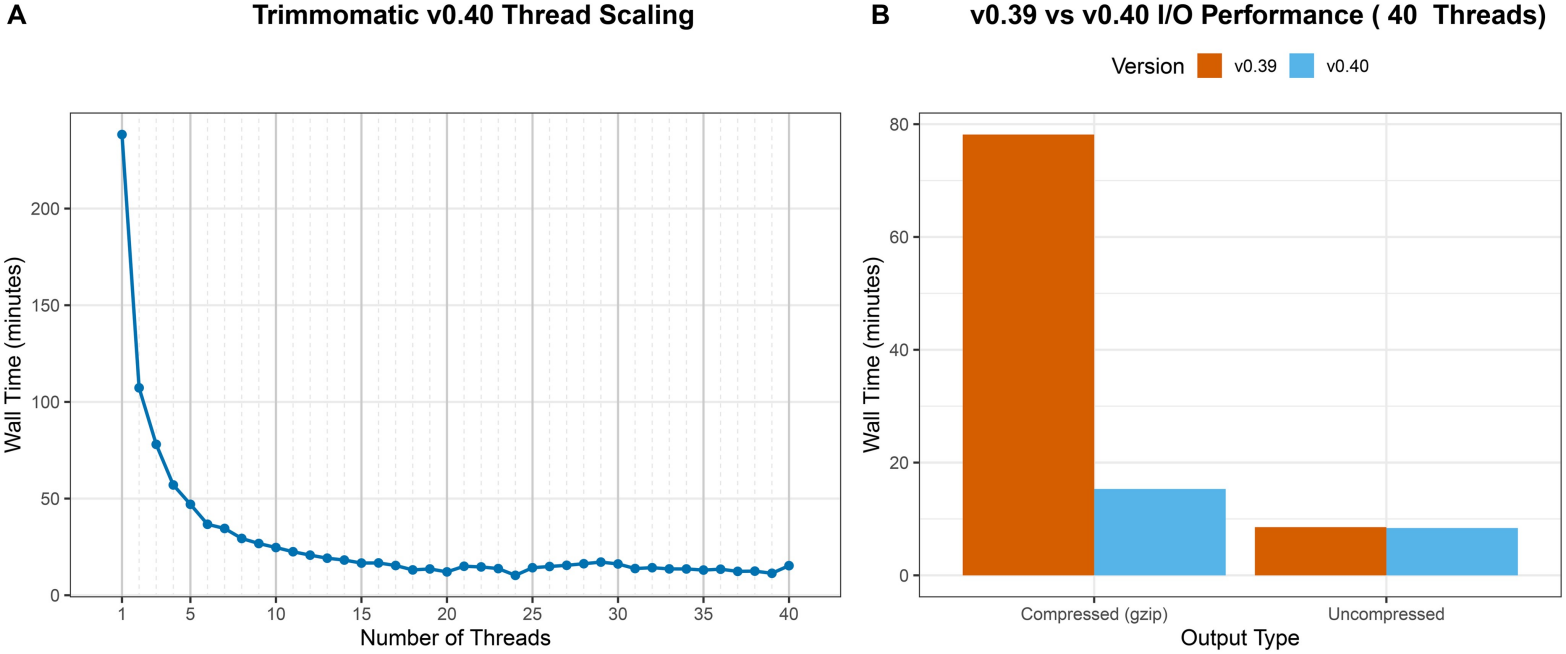
Performance scaling and I/O optimization in Trimmomatic v0.40. (A) Thread scaling behavior. Wall-clock execution time (minutes) is plotted as a function of allocated threads (1–40) using an 18.1 Gb Manihot esculenta whole-genome sequencing dataset (∼184.7 million read pairs). Processing included standard adapter clipping and quality filtering. (B) Impact of parallel compression at high core counts. Wall-clock execution times are compared between Trimmomatic v0.39 (single-threaded compression) and v0.40 (parallel compression) when executing with 40 threads. Results are shown for both standard compressed (gzipped) and uncompressed output generation, illustrating the resolution of the GZIP writing bottleneck in the updated architecture.

First, to demonstrate thread scaling, the latest version (v0.40) was run on a 18.1 Gb WGS dataset (SRA Run: ERR15075373), containing 184.7 million read pairs from *Manihot esculenta*, sequenced on an Illumina NovaSeq 6000 (Beier *et al.* 2025). The experiment was run on a 40-core server with 125 GB of RAM. A typical trimming command, engaging both adapter clipping and quality filtering, was executed while varying the thread count (-threads parameter) from 1 to 40. The command used was: ILLUMINACLIP:TruSeq3-PE-2.fa:2:30:10 SLIDINGWINDOW:4:20 MINLEN:36. The results show a significant initial speedup that quickly reaches a point of diminishing returns (Fig. 1A). The wall time was reduced from 238 minutes (on a single thread) to a performance plateau that begins at approximately 20 threads. A minimum runtime of 13.9 minutes was observed at 24 threads (a 17.1-fold speedup), with performance remaining relatively flat up to 40 threads, indicating that for this dataset, parallel overhead from thread management and resource contention fully balances the benefits of additional cores beyond this point.

Second, to isolate the benefit of parallel compression, we compared v0.39 (which includes multithreading but not parallel compression) with v0.40 (which adds parallel compression). Both versions were run with 40 threads on the same dataset (Fig. 1B). When writing gzipped output, v0.40 was 5.1-fold faster than v0.39 (15.3 minutes vs 78.2 minutes). This demonstrates that for high-core-count jobs, standard GZIP compression in v0.39 had become the primary bottleneck, and this bottleneck is resolved by the parallel compression in v0.40. This gain was eliminated when writing uncompressed output (8.39 minutes for v0.40 vs 8.55 minutes for v0.39), confirming that the parallel compression algorithm is the source of the speedup. A compression ratio analysis comparing the parallel output (40 threads) to standard single-threaded compression (1 thread) revealed a practically negligible penalty on file size. On the Manihot dataset, the parallel implementation increased the output file sizes by less than 0.002% (an addition of 159 KB to the 11.9 GB Read 1 file, and 142 KB to the 12.3 GB Read 2 file). This minor increase is an expected trade-off for block-based parallel compression algorithms, as the compression dictionary must be reset between blocks to allow independent processing.

Finally, to contextualize the performance of Trimmomatic v0.40 within the broader landscape of read-preprocessing software, a thorough benchmark analysis was performed against five widely used modern tools: fastp (Chen 2025), RabbitTrim (Wang *et al*. 2025), Cutadapt (Martin 2011), Skewer (Jiang *et al*. 2014), and BBDuk (Bushnell 2014). This evaluation, which assesses scaling behavior up to 240 threads, peak memory footprint, and empirical trimming accuracy based on residual adapter quantification across both plant and human datasets, is detailed in the Supplementary Data.

## Conclusion

Trimmomatic has evolved substantially from its original release to meet the modern challenges of NGS data analysis. Through the implementation of multithreading, parallel compression, and a more versatile set of trimming tools, it remains a state-of-the-art solution for read preprocessing. Enhancements to usability and a focus on code sustainability ensure that Trimmomatic will be a reliable and essential component of bioinformatics research.

## Supplementary Material

btag331_Supplementary_Data

## Data Availability

The genomic data used in this study is published through the EMBL-EBI European Nucleotide Archive (ENA) under BioProject accession number PRJEB89494, in particular SRA Run: ERR15075373.
