# Supplementary material for: Trimmomatic: a decade of feature-rich, high-performance NGS read preprocessing
Source: Bioinformatics. 2026 May 22;42(6):btag331. doi: 10.1093/bioinformatics/btag331 (PMC13242794; doi:10.1093/bioinformatics/btag331)
Supplement: btag331_Supplementary_Data [file btag331_supplementary_data.zip › 2026-05-11_SupplementaryData_Trimmomatic.pdf]

Supplementary Data

Comprehensive performance, accuracy, and scalability benchmark

To evaluate the overall efficiency and accuracy of Trimmomatic v0.40, we conducted a broad benchmark against five widely used read-preprocessing tools: RabbitTrim (v2.0.0), fastp (v1.1.0), BBDuk (v39.52), Skewer (v0.2.2), and Cutadapt (v5.2). Testing was performed on a high-performance compute node equipped with dual AMD EPYC 64-core processors, 256 GB RAM, and NVMe solid-state storage. We utilized two primary datasets: a complex *Manihot esculenta* plant dataset (~78 million read pairs, 2x150 bp) and a high-quality *Homo sapiens* human from Genome-In-A-Bottle project (HG001) dataset (~20 million read pairs, 2x150 bp). To ensure a comparable evaluation of computational efficiency, output compression levels were standardized to level 6 (the default for standard gzip and Trimmomatic) where supported by the tool's command-line interface. Furthermore, to establish a standardized baseline for both accuracy and speed, all evaluated tools were supplied with identical adapter sequence information *a priori.*

Standard accuracy metrics based on simulated data often fail to capture the complexities of empirical sequencing libraries. Therefore, we measured accuracy by quantifying the number of true residual adapter sequences left in the datasets after trimming. Output reads were exhaustively scanned for the 13-bp Illumina TruSeq Universal Adapter seed (AGATCGGAAGAGC). To distinguish true adapter read-throughs from random genomic matches, we applied a strict "seed-and-extend" verification algorithm: the sequence immediately following the seed was required to match the known adapter extension signatures for Read 1 or Read 2, allowing for one mismatch. Matches occurring at the extreme 5' end of the read (positions <15) were excluded as likely artifacts.

Trimmomatic's palindrome-based trimming algorithm demonstrated the highest accuracy. On the Plant dataset, Trimmomatic left only 14 true residual adapter reads. RabbitTrim, which implements the same algorithm, achieved identical accuracy. In contrast, standard overlap-detection approaches left substantially more residuals: fastp (810), BBDuk (3,847), Skewer (14,900), and Cutadapt (23,874). On the Human dataset, Trimmomatic and RabbitTrim left 0 residuals, while fastp left 17.

We conducted a scaling stress test allocating 1, 2, 4, 8, 16, 20, 40, 80, 160, and 240 threads. At an allocation of 40 threads, Trimmomatic v0.40 processed the complex Plant dataset in 10.9 minutes, operating at speeds comparable to RabbitTrim (11.4 minutes) and outperforming fastp (22.0 minutes). As a trade-off for its Java-based architecture and multi-threaded I/O queues, Trimmomatic utilized a peak memory footprint between 15 and 25 GB, whereas C++ implementations like fastp required approximately 2–3 GB.

Regarding scalability, execution times for all tested tools generally plateaued around 64 threads, indicating hardware or I/O saturation. We noted that both fastp and RabbitTrim enforce hard-coded concurrency limits at 64 and 63 threads, respectively; allocating resources beyond these limits yielded no changes in execution behavior. Trimmomatic v0.40 imposes

no such limits, maintaining stable execution up to 240 threads, though practical speed gains beyond the 64-thread threshold remain marginal for all tools (Supplementary Figure S1).

To further assess storage optimization, a comparative analysis was conducted on the compressed output file sizes across all tools at both low (8) and high (40) thread levels (see Supplementary Table S1). While the majority of the tools exhibited stable file sizes across varying thread counts, this analysis exposed a significant difference in RabbitTrim's execution based on thread number.

Supplementary Table S1: Comparison of output file sizes and compression consistency.

Tool	Threads	Plant R1 (MB)	Plant R2 (MB)	Human R1 (MB)	Human R2 (MB)
Trimmomatic	8	11,439.7	11,750.9	3,259.8	3,431.7
Trimmomatic	40	11,439.7	11,750.9	3,259.8	3,431.7
RabbitTrim	8	14,763.4	15,180.9	4,067.9	4,270.1
RabbitTrim	40	11,418.1	11,728.9	3,255.8	3,428.0
fastp	8	12,475.5	13,063.7	3,566.8	3,952.8
fastp	40	12,475.5	13,063.7	3,566.8	3,952.8
Cutadapt	8	12,280.7	12,877.2	3,768.7	4,171.0
Cutadapt	40	12,280.7	12,877.2	3,768.7	4,171.0
BBDuk	8	12,579.3	13,181.7	3,800.4	4,217.5
BBDuk	40	12,584.8	13,187.2	3,802.1	4,219.0
skewer	8	12,837.1	13,438.6	3,893.6	4,287.3
skewer	40	12,837.1	13,438.6	3,893.6	4,287.3

Investigating the output with the "file" command revealed that at lower thread counts (1–20 threads), RabbitTrim quietly reverts to "max speed" compression, disregarding the user-specified "--compressLevel 6" flag. Consequently, the output files are 23–29% larger than those produced at high thread counts (40 and more threads). At these levels, the tool utilizes the pigz library for parallel compression and adheres to the compressLevel flag. A fair comparison between RabbitTrim and the other tools is therefore impeded at these lower thread counts, as also the CPU workload and runtime are affected by this inconsistency (Supplementary Figure S1).

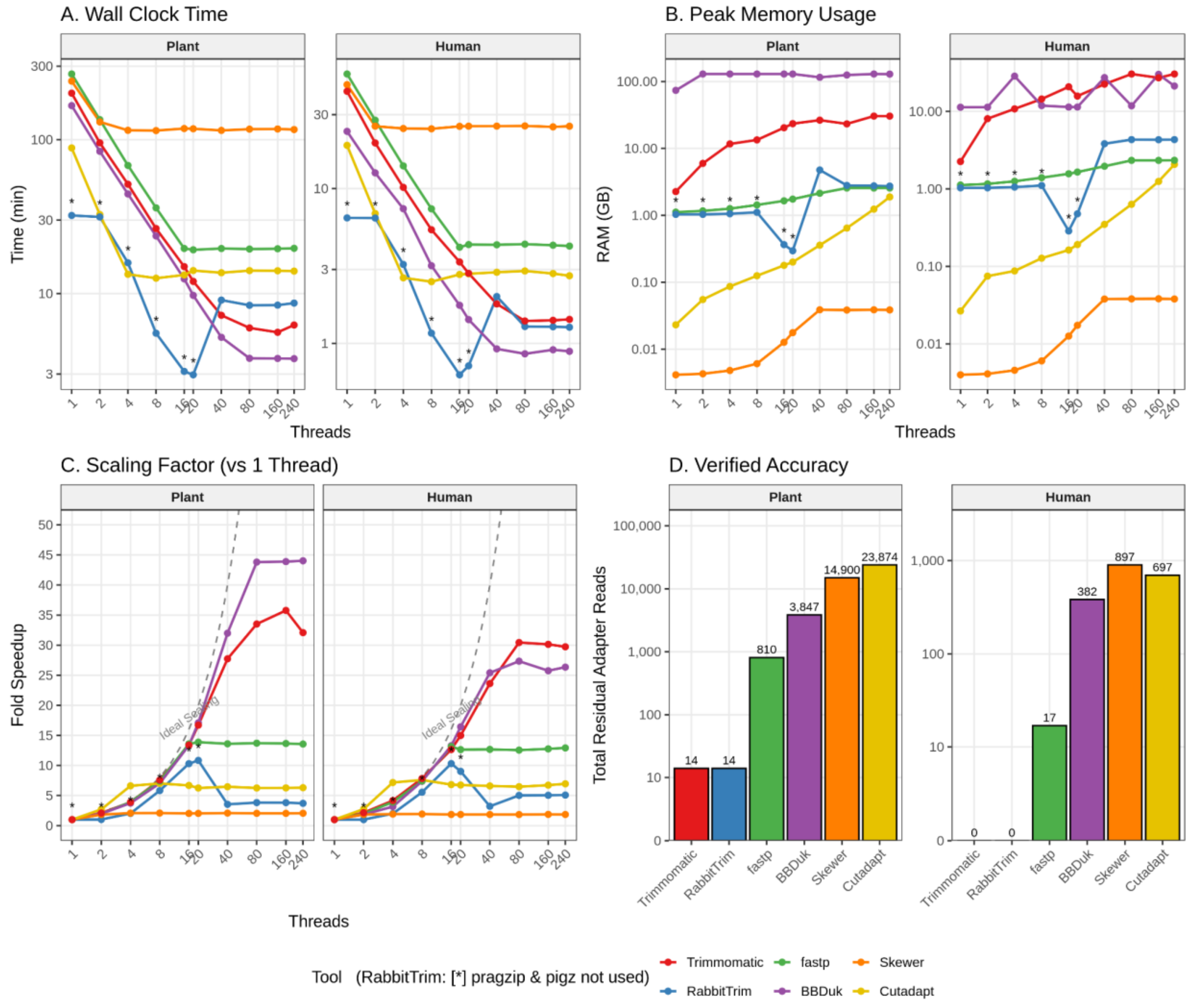

Supplementary Figure S1. Comprehensive performance, scalability, and accuracy benchmark of read-preprocessing tools. Evaluation of Trimmomatic v0.40 against five other tools (RabbitTrim, fastp, BBDuk, Skewer, and Cutadapt) across a *Manihot esculenta* (Plant) dataset and a *Homo sapiens* (Human) dataset. **(A)** Wall-clock execution time (minutes) plotted on a log10 scale across thread allocations ranging from 1 to 240 (x-axis, log2 scale). **(B)** Peak memory consumption, measured as Maximum Resident Set Size (GB) on a log10 scale. **(C)** Scaling efficiency, calculated as the fold speedup relative to each tool's baseline execution at 1 threads. The dashed grey line indicates the theoretical ideal linear scaling trajectory. **(D)** Verified trimming accuracy, displaying the total number of true residual adapter sequences remaining in the processed reads, plotted on a pseudo-log10 scale (lower values indicate higher accuracy). Where supported by the command-line interface, output compression was standardized to level 6 for all tools. Asterisks (*) on the RabbitTrim trajectory indicate specific thread configurations where its adaptive engine dynamically reverted to single-threaded standard zlib decompression and compression (disabling both pragzip and pigz). Notably this adaptive engine reverted to “max speed” compression, resulting in significantly larger output files when compared to larger thread counts and simultaneously reduced computational workload.

Evaluation of trimming throughput with uncompressed data

To isolate the computational performance of the underlying trimming algorithms from the overhead of GZIP compression, we benchmarked all tools using uncompressed FASTQ output. This scenario is particularly relevant for bioinformatics pipelines that utilize streaming workflows, where outputs are piped directly into downstream applications without intermediate storage.

To ensure a fair comparison and avoid hardware-level I/O bottlenecks (disk write saturation) that occur when writing large uncompressed files at high thread counts, these tests were standardized at a fixed allocation of 4 threads. This configuration represents a typical real-world scenario for preprocessing in a multi-stage pipeline (Supplementary Table S2).

Supplementary Table S2: Execution time for uncompressed output at 4 threads.

Tool	Plant - Time (min)	Human - Time (min)
Cutadapt	7.7	1.5
RabbitTrim	10.9	2.2
Trimmomatic	16.3	2.7
BBDuk	43.7	6.5
Skewer	62.0	12.2
fastp	67.8	13.9

When the GZIP compression bottleneck is removed, Trimmomatic v0.40 demonstrates good raw throughput. While the C++ and Python/C-based tools Cutadapt and RabbitTrim lead in absolute speed, Trimmomatic v0.40 processes the datasets significantly faster than other widely used tools such as BBDuk, Skewer, and fastp. These results show that Trimmomatic's core engine is efficient for high-speed streaming applications and low-core cloud environments.

Direct comparison on high-coverage human data

To further contextualize performance against RabbitTrim—a highly optimized C++ implementation of the Trimmomatic algorithm—we conducted a targeted evaluation using a specific high-coverage human dataset (SRR7890824, ~410 GB uncompressed).

When standardizing the output compression to Level 6 to ensure equivalent I/O workloads, the execution times of the two tools converged closely. For example, at an allocation of 64 threads, RabbitTrim completed the preprocessing in approximately 18 minutes, while Trimmomatic v0.40 completed it in 21 minutes.

Log analysis of the execution runs indicated differences in thread management. RabbitTrim utilizes an adaptive thread allocation system that dynamically assigns threads based on runtime factors. Under certain thread configurations during our testing, this engine was observed to revert to single-threaded decompression (pragzip) or single-threaded compression (pigz). Conversely, Trimmomatic v0.40 leverages standard Java parallel streams, yielding predictable, multi-threaded compression behavior across varying core allocations without requiring external native dependencies (Supplementary Figure S2).

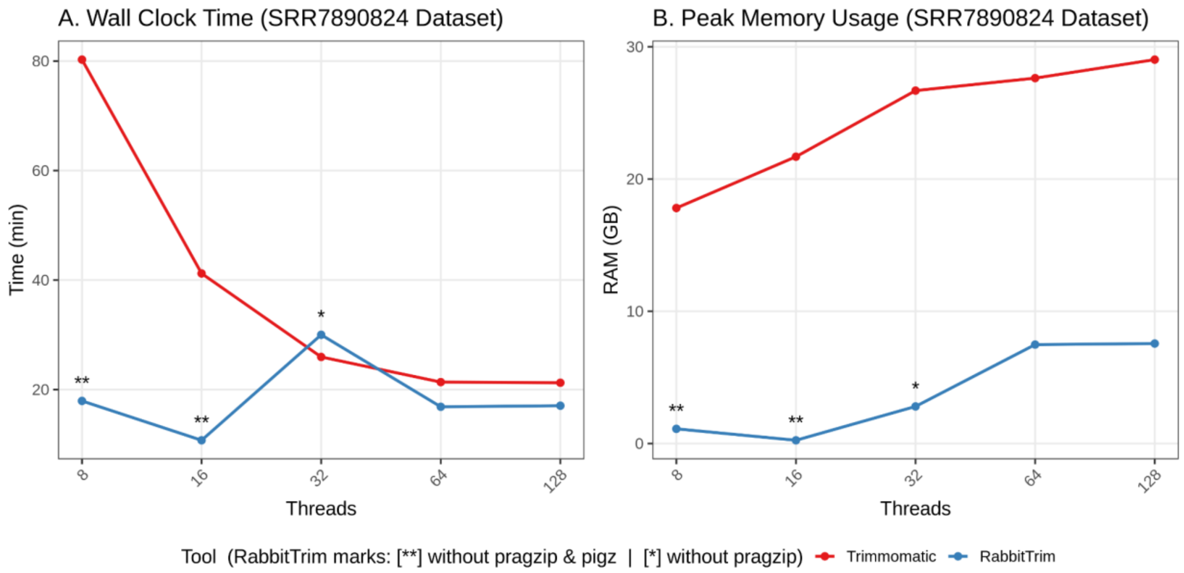

Supplementary Figure S2. Direct performance and memory comparison of Trimmomatic v0.40 and RabbitTrim on high-coverage human sequencing data. Preprocessing performance on the ~410 GB SRR7890824 human dataset, processed with a standardized output compression level of 6 to ensure equivalent I/O workloads. **(A)** Wall-clock execution time (minutes) across 8 to 128 threads (x-axis, log2 scale). **(B)** Peak memory usage (GB). Annotations denote instances where RabbitTrim's adaptive engine altered its parallel I/O components: [**] indicates neither multi-threaded decompression (pragzip) nor multi-threaded compression (pigz) was utilized; [*] indicates only multi-threaded decompression (pragzip) was disabled. Notably this also reverted to "max speed" compression, resulting in significantly larger output files when compared to larger thread counts and simultaneously reduced computational workload.

Evaluation of fastp versions and adapter detection parameters

In practice, many users of fastp utilize its internal adapter auto-detection algorithm rather than explicitly providing known adapter sequences via the command line. Furthermore, recent updates to fastp (v1.2.0 and beyond) have introduced parallel compression, mirroring the architectural improvements Trimmomatic made from v0.39 to v0.40. To explore these scenarios and software changes, we conducted a comparison of Trimmomatic v0.40 against four distinct fastp configurations: fastp v1.1.0 (the version utilized in our primary benchmark) and fastp v1.3.3, both executed with and without explicitly provided adapter sequences (via the --fasta_adapter flag).

As illustrated in **Supplementary Figure S3**, updating to fastp v1.3.3 yields notable improvements in wall-clock execution time compared to v1.1.0, primarily driven by the integration of parallel compression. Furthermore, omitting the explicit FASTA adapter input and relying exclusively on fastp's internal adapter detection routines provides a slight additional speed increase.

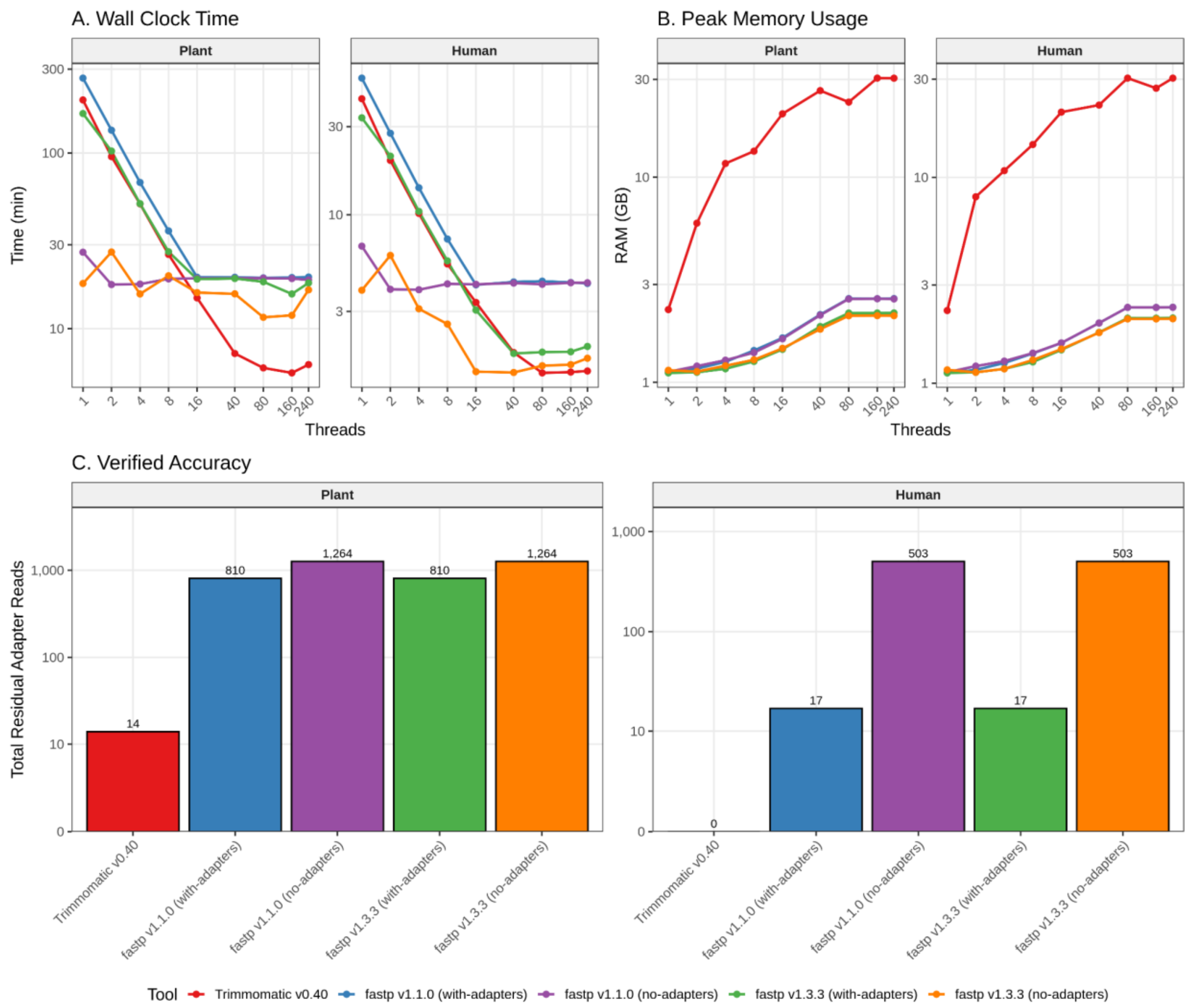

Supplementary Figure S3. Performance, memory, and accuracy evaluation of fastp versions and adapter detection parameters. Comparison of Trimmomatic v0.40 against fastp versions 1.1.0 and 1.3.3 (both executed with and without explicit adapter sequences provided via the --fasta_adapter flag) across a *Manihot esculenta* (Plant) dataset and a *Homo sapiens* (Human) dataset. **(A)** Wall-clock execution time (minutes) plotted on a log10 scale across thread allocations ranging from 1 to 240 (x-axis, log2 scale). **(B)** Peak memory consumption, measured as Maximum Resident Set Size (GB) on a log10 scale. **(C)** Verified trimming accuracy, displaying the total number of true residual adapter sequences remaining in the processed reads, plotted on a pseudo-log10 scale (lower values indicate higher accuracy). Output compression was standardized to level 6 across all configurations. Notably, the data point for 20 threads was excluded from this analysis, as fastp v1.3.3 failed to terminate at this specific thread count due to probably a software bug.

Data availability

For full transparency and reproducibility, all bash scripts, execution logs, verification algorithms, and data visualization code generated during these benchmarks have been deposited in a public GitHub repository: https://github.com/usadellab/trimmomatic-validation-2026.
