# Supplementary material for: Trimmomatic: a decade of feature-rich, high-performance NGS read preprocessing
Source: Bioinformatics. 2026 May 22;42(6):btag331. doi: 10.1093/bioinformatics/btag331 (PMC13242794; doi:10.1093/bioinformatics/btag331)
Supplement: btag331_Supplementary_Data [file btag331_supplementary_data.zip › 260320_Figure_Scaling_Benchmark.pdf]

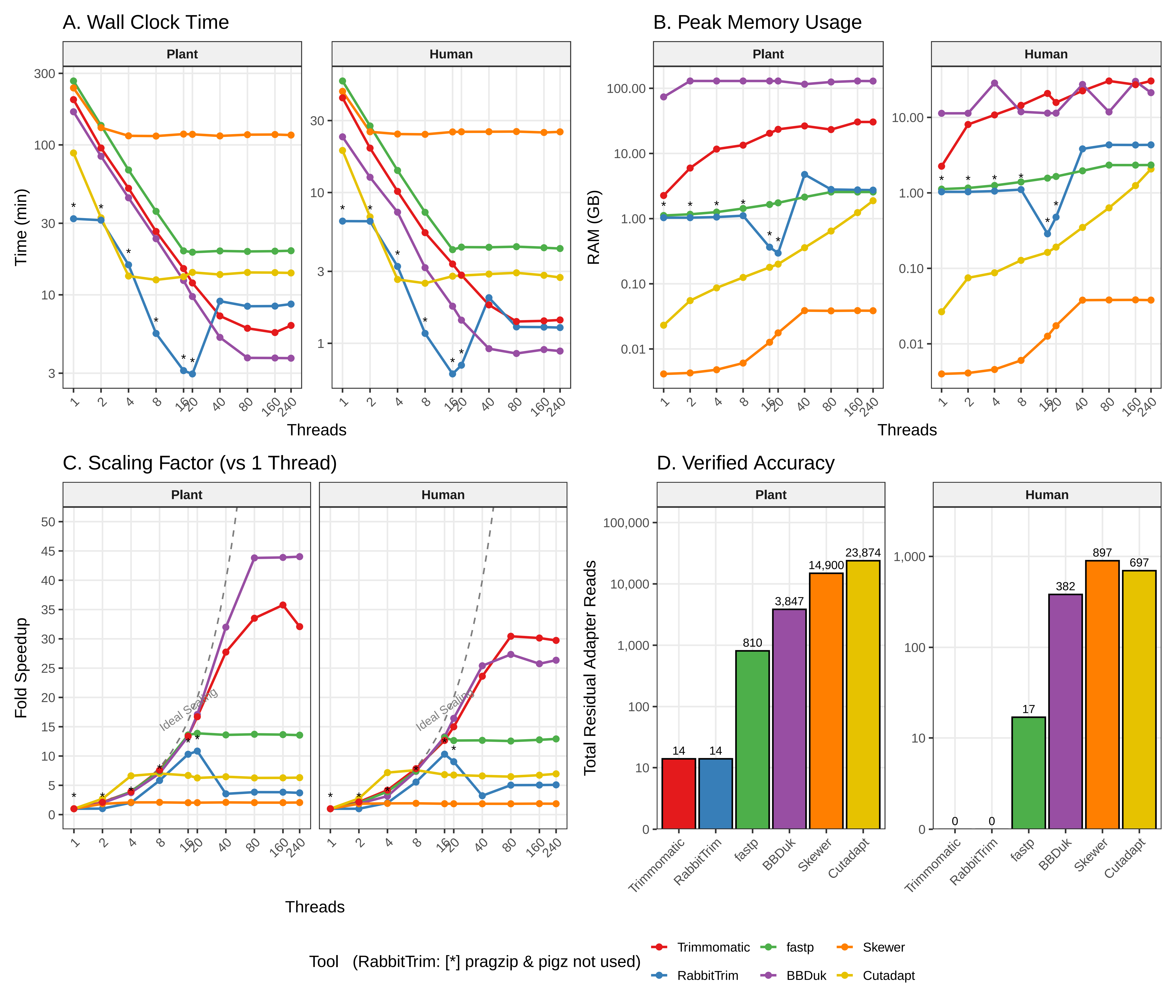
A. Wall Clock Time
Plant
Human
Time (min)
300
100
30
10
3
1
Threads
1 2 4 8 16 20 40 80 160 240
B. Peak Memory Usage
RAM (GB)
100.00
10.00
1.00
0.10
0.01
C. Scaling Factor (vs 1 Thread)
Fold Speedup
50 45 40 35 30 25 20 15 10 5 0
Ideal Scaling
D. Verified Accuracy
Total Residual Adapter Reads
100,000
10,000
1,000
100
10
0
14
14
810
3,847
14,900
23,874
0
0
17
382
897
697
Trimmomatic
RabbitTrim
fastp
BBDuk
Skewer
Cutadapt
Tool (RabbitTrim: [*] pragzip & pigz not used)
