# Supplementary material for: Trimmomatic: a decade of feature-rich, high-performance NGS read preprocessing
Source: Bioinformatics. 2026 May 22;42(6):btag331. doi: 10.1093/bioinformatics/btag331 (PMC13242794; doi:10.1093/bioinformatics/btag331)
Supplement: btag331_Supplementary_Data [file btag331_supplementary_data.zip › Figure_RabbitTrim_Dataset_Benchmark.pdf]

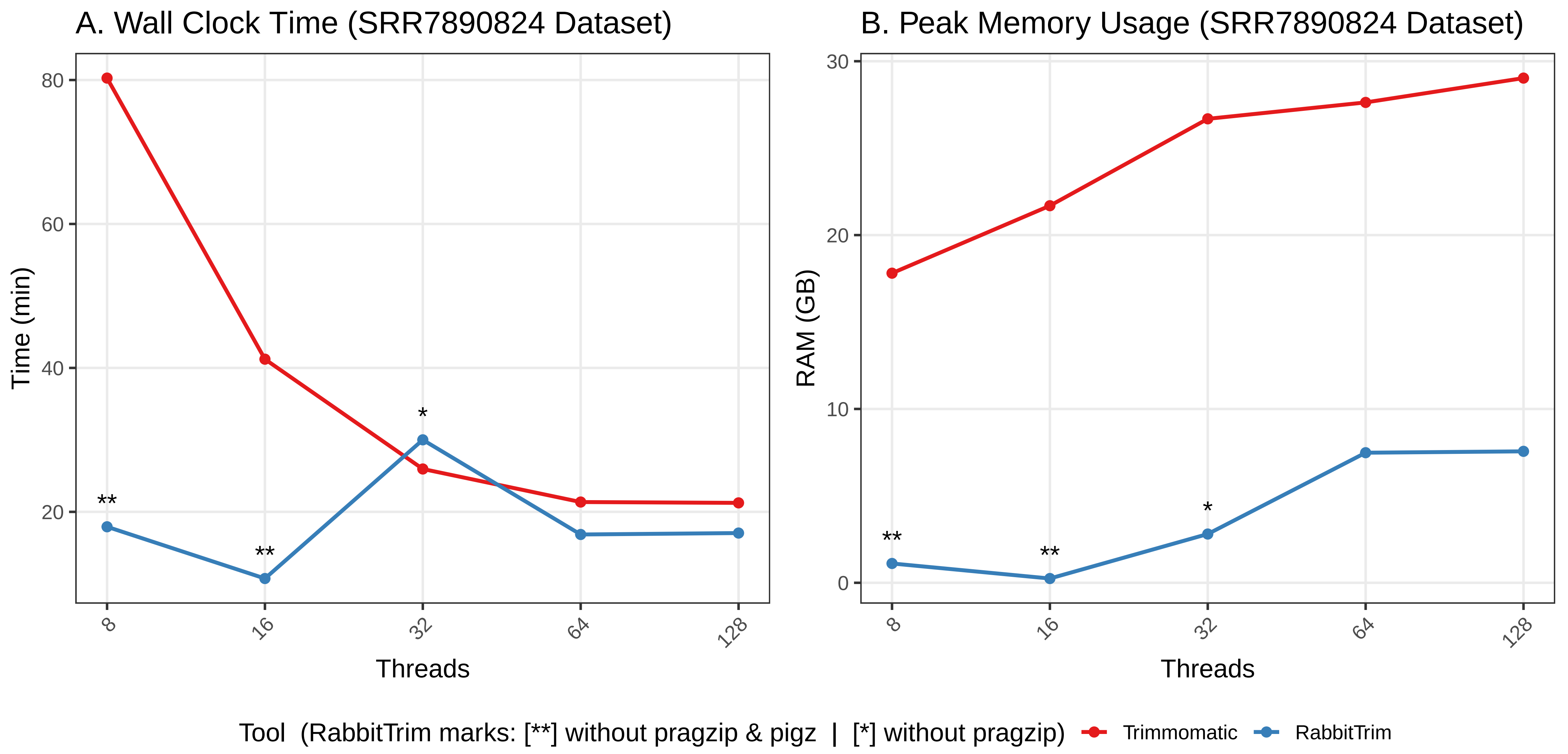

A. Wall Clock Time (SRR7890824 Dataset)
B. Peak Memory Usage (SRR7890824 Dataset)
Time (min)
80
60
40
20
**
**
*
8
16
32
64
128
Threads
RAM (GB)
30
20
10
0
**
**
*
8
16
32
64
128
Threads
Tool (RabbitTrim marks: [**] without pragzip & pigz | [*] without pragzip)
Trimmomatic
RabbitTrim
