# Supplementary figures and images for: Trimmomatic: a decade of feature-rich, high-performance NGS read preprocessing
Source: Bioinformatics. 2026 May 22;42(6):btag331. doi: 10.1093/bioinformatics/btag331 (PMC13242794; doi:10.1093/bioinformatics/btag331)

A. Wall Clock Time

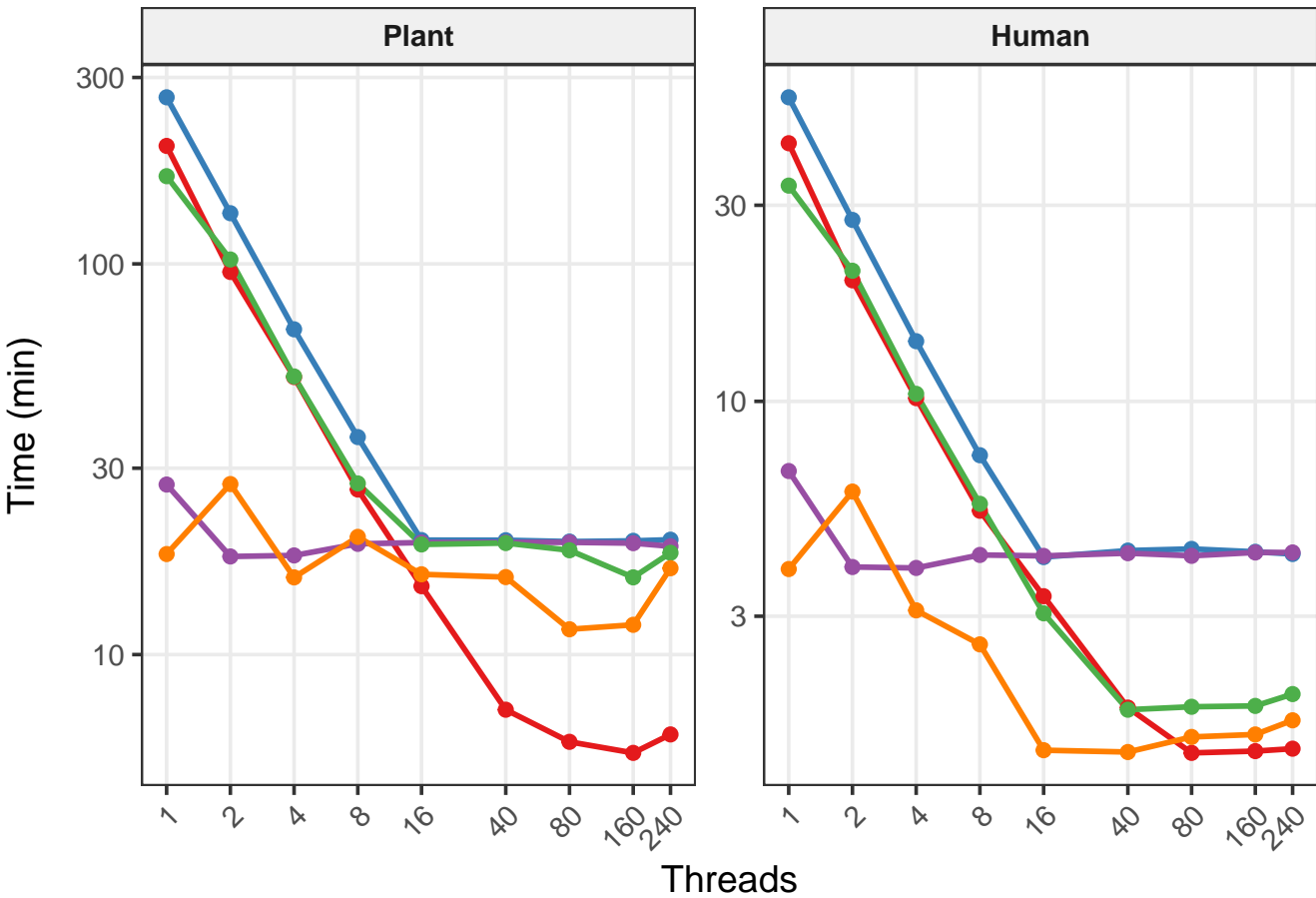

B. Peak Memory Usage

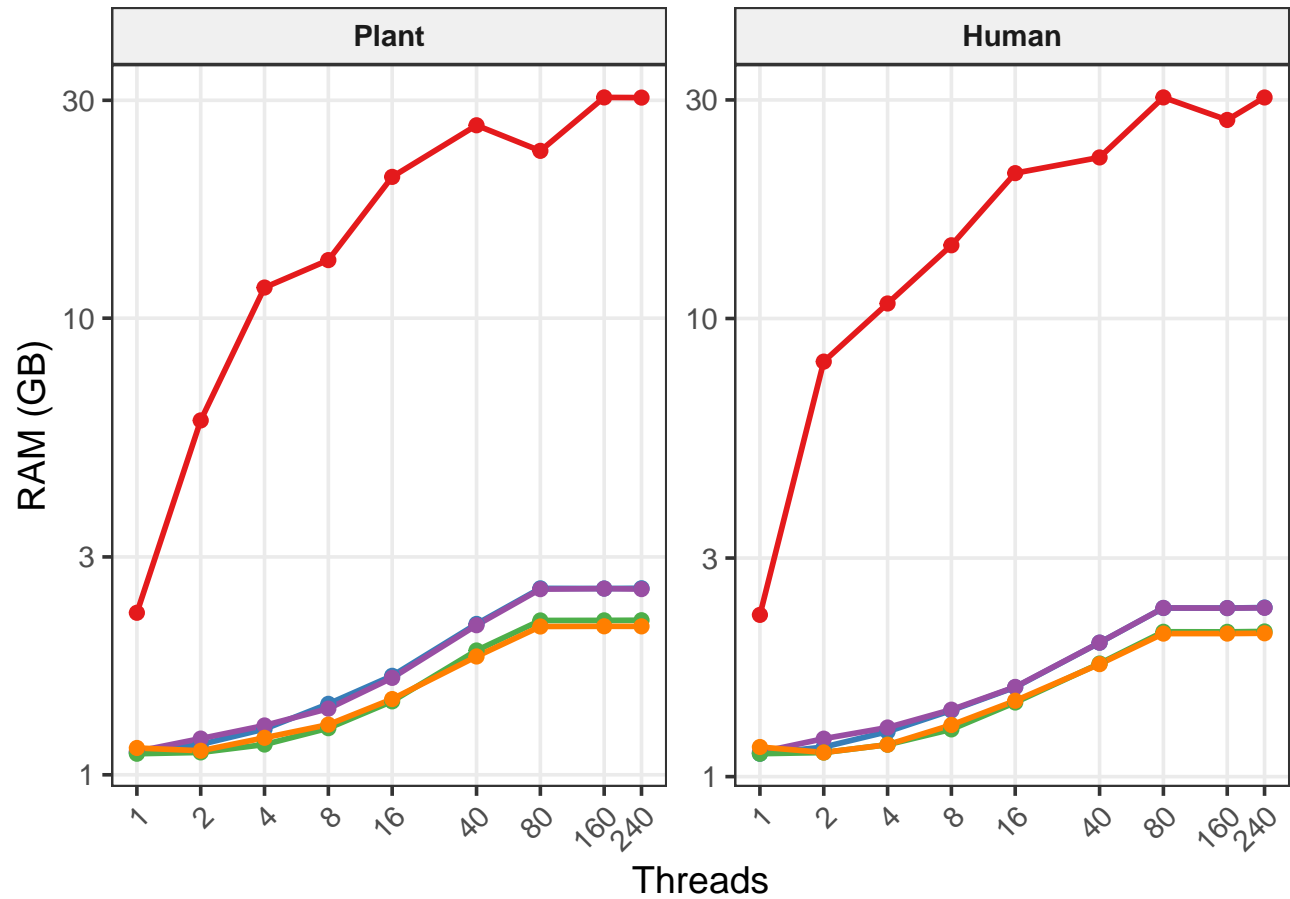

C. Verified Accuracy

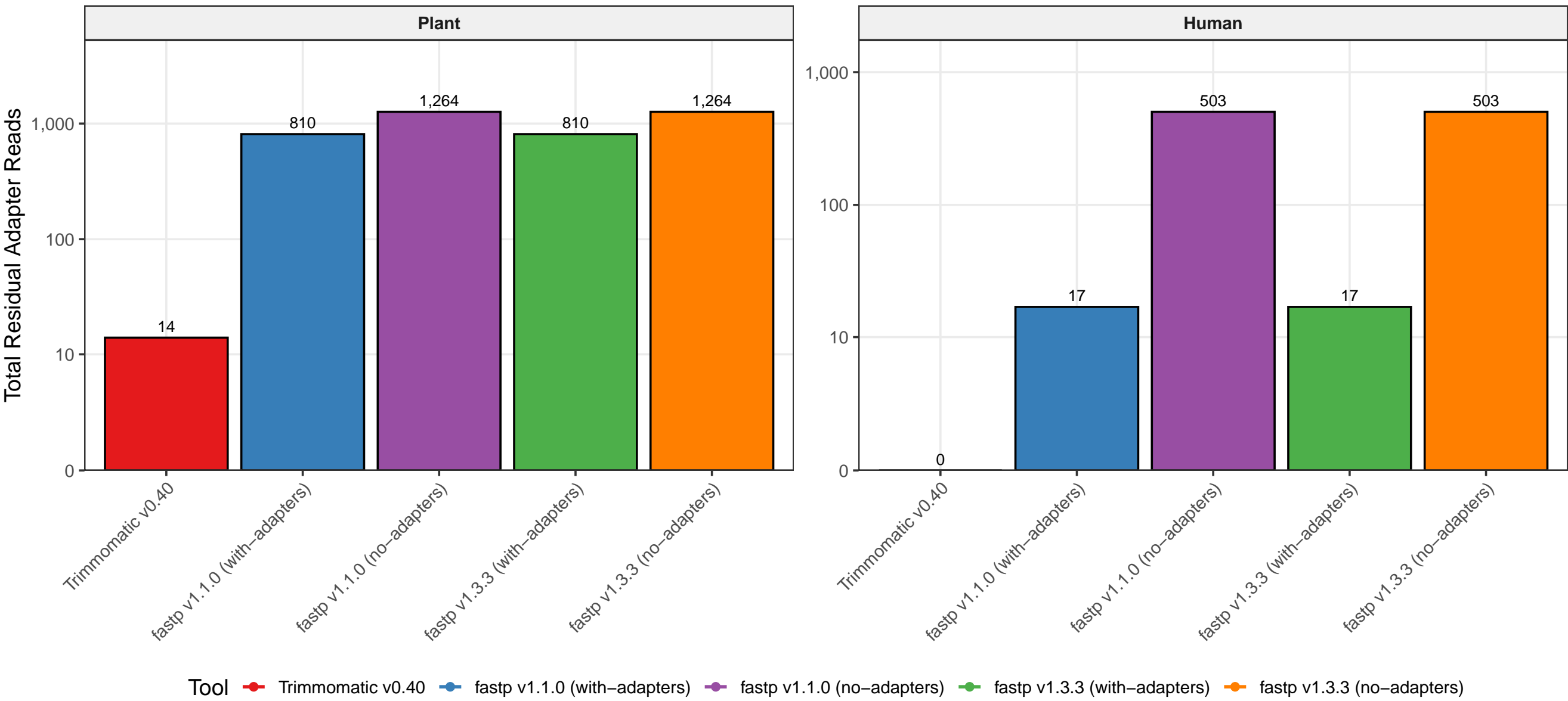

Supplement: btag331_Supplementary_Data [file btag331_supplementary_data.zip › Figure_Fastp_Comparison.pdf]
